# Is memantine a potential therapeutic for Rett syndrome?

**DOI:** 10.3389/fnins.2013.00245

**Published:** 2013-12-17

**Authors:** Olivia Bello, Kelsey Blair, Christopher Chapleau, Jennifer L. Larimore

**Affiliations:** ^1^Department of Biology, Agnes Scott CollegeDecatur, GA, USA; ^2^Department of Pharmacy, University of Alabama at BirminghamBirmingham, AL, USA

**Keywords:** memantine, down syndrome, Rett syndrome, Fragile X syndrome, Alzheimer's disease

## Abstract

Memantine is a low-affinity, voltage-dependent, non-competitive N-methyl-D-aspartate (NMDA) receptor antagonist. It is classified as a neuroprotective aminoadamantane. It does not cure or reverse Alzheimer's but it does effectively treat symptoms, slows the progression of the disease and allows many patients to perform daily cognitive activities with clear thoughts. Based on it's success in patients with Alzheimer's, memantine has been tested in other neurological disorders with impaired learning and memory. In this review, we will discuss the success and failures of memantine in Downs Syndrome and Fragile X research and from those results, assess the potential benefit of memantine in Rett Syndrome (RTT).

## Introduction

Memantine was first synthesized in 1968 as a derivative of amantadine, an anti-influenza agent (Lipton, 2004). It took nearly 15 years for scientists to discover that memantine was not dopaminergic or anti-cholinergic but in fact a non-competitive N-methyl-D-aspartate (NMDA) receptor antagonist. Proper NMDA function is required for synaptic plasticity. Compared to other NMDA receptor channel blockers such as ketamine and phencyclidine (PCP), memantine has a lower affinity for the receptor (Zdanys and Tampi, 2008). Memantine inhibits the Ca^2+^ influx by binding the NMDA receptor with higher affinity compared to Mg^2+^ ions. Its low receptor affinity allows for the preservation of the physiological function of the receptor. The receptor can be activated by high concentrations of glutamate released after depolarization of the presynaptic neuron (Hosenbocus and Chahal, 2013).

Memantine is one of the primary pharmacological therapies for Alzheimer's disease. From an anatomical point of view, Alzheimer's disease distinguishes itself from other neurological diseases in that the pathology is associated with atrophy in areas of the brain important for learning and memory which is a direct result of the molecular hallmarks of the disease: including the development of amyloid plaques, tau hyper-phosphorylation, and neurofibrillary tangles. For the most part, these three events are believed to cause cerebral atrophy by destroying synapse and dendrites, thus leading to the destruction of the entire neuron. Current pharmacological therapy including drugs such as donepezil, galantamine, or rivastigmine, which are classified as cholinesterase inhibitors, in addition to memantine, are believed to slow down these degenerative processes in the brain by altering calcium uptake into the neuron, or increasing the synaptic concentration of acetylcholine (Cummings et al., 2002; Rogawski and Wenk, 2003).

Memantine acts as a neuroprotectant by decreasing glutamate excitotoxicity and has been known to increase levels of brain-derived neurotrophic factor (BDNF) thus influencing synaptic plasticity in rats, β-amyloid-induced apoptotic death and neuroinflammation in the hippocampus (Picada et al., 2011). In the Morris water maze, memantine improved acquisition performance, spatial accuracy, and increased durability of synaptic plasticity (Sahiner et al., 2011). Over the past decade, memantine drug trial research has been conducted in several neurological disorders with cognitive disabilities including Down Syndrome (DS), Fragile X Syndrome (FXS), and Rett Syndrome (RTT). This review will focus on their results and future potential for treatment in patients with RTT.

## Down syndrome and memantine

DS is the most common chromosome abnormality in humans caused by the trisomy of human chromosome 21. DS is most known for its effect on neurodevelopment but it also has a neurodegenerative component, which includes neuropathology indistinguishable from Alzheimer's disease (Costa et al., 2008). Nearly 40% of patients with DS over the age of 60 years also have a diagnosis of dementia (Hanney et al., 2012). For patients with DS, Alzheimer's is the most common form of dementia (Mohan et al., 2009). The gene Down Syndrome Critical Region 1 (DSCR1), a chromosome 21 gene, has been thought to cause Alzheimer's-like pathology. The Ts65Dn mouse model of DS happens to have three copies of the mouse ortholog DSCR1 (Costa et al., 2008). The main pathological features of DS and the increased risk of dementia are caused by the trisomy of chromosome 21 and Alzheimer specific risk alleles, potentially involving the DSCR1 gene. This genotype gives way to an increase in amyloid β and hyper-phosphorylation of tau tangles (Hanney et al., 2012). It is important to note that people with DS overproduce amyloid β because of gene dysregulation many of which are not linked to Alzheimer's.

### Efficacy of memantine on Ts65Dn mice

Calcineurin (CaN) is a phosphatase that interacts with DSCR1 and its phosphatase activity is inhibited by loss of DSCR1 in DS, which alters the NMDA receptor kinetics. Memantine has produced changes on NMDA receptor kinetics that mimics CaN, strongly suggesting a possible rescue of altered NMDA kinetics in DS. In another study, memantine partially restored the physiological function of the NMDA receptor and improved learning and memory in mice (Costa et al., 2008). Three significant findings emerged from this study, the first being that acute memantine injections selectively improved the behavioral performance of 4–6 months old Ts65Dn mice in a contextual fear conditioning paradigm. Secondly, a memantine injection prior to the fear conditioning session was found to be necessary in order to produce memory enhancement in the 4–6 months old Ts65Dn mice. Lastly, to determine if there was an age dependence on the affects demonstrated, acute memantine injections selectively improved the performance of 10–14 months old Ts65Dn mice. The conclusion of this study demonstrates that acute memantine injections have the ability to rescue performance deficits in the Ts65Dn mouse model.

### Memantine effects in patients with down syndrome

Human drug trials in patients with DS were published in 2012 (Hanney et al., 2012). The study researched the effects of memantine on both patients over the age of 40 with DS and patients of any age with DS and dementia. This double blind, randomized drug trial ran for 1 year in Norway and the United Kingdom. Unfortunately the benefits found in the mouse model research were not translated into people with DS. Cognitive decline persisted throughout the entire study in both patient groups.

The study did not find memantine to be useful, however drug trials should not stop here. The research of Hanney et al. thus far has not looked specifically at moderate to severe cases in patients with DS and dementia. The patient groups they worked with were patients over the age of 40 with DS and patients of any age with DS and either diagnosed or undiagnosed dementia. Future researchers should use a clearly defined patient pool with similar age diversity and include both moderate and severe cases of dementia in patients with DS. In the future, larger cohort groups need to be considered and further testing of various aspects of the drug such as a study on cholinesterase inhibitors will push memantine trials further. Studies should also address if mental decline can be prevented by early administration of memantine.

## Fragile X syndrome and memantine

FXS is the most common single gene inherited cause of intellectual disability among boys. FXS has a cysteine-guanine-guanine (CGG) trinucleotide repeat in the fragile X mental retardation 1 gene (FMR1) located near the long arm of the X chromosome (Erickson et al., 2009). Memantine is an appealing drug candidate for FXS treatment because FXS pathophysiology involves glutamate receptor dysfunction. Since memantine is a noncompetitive antagonist at glutamate receptors, it has the potential to aid symptoms associated with FXS.

### Fragile X syndrome memantine treatment in mice

Studies by Wei et al. on the FMR1 knockout mouse model, the current model for FXS and investigated memantine's effect on cerebellar granule cells (CGCs) function. Cell adhesion assay and cell migration assay as well as immunofluorescence were used in this study to assess memantine's effects on cells in culture. The results revealed that memantine promoted CGCs adhesion, had little effect on CGCs migration, restored dendritic spines to normal levels, stimulated synapse formation and restored excitatory synapses to a normal range (Wei et al., 2012). One of the most interesting results is that memantine was able to restore the mushroom spine density levels in the knockout mice to normal. This property of memantine is similar to BDNF, which also aids in the restoration of spines back to normal (Luine and Frankfurt, 2012). The results of memantine on the CGCs are promising and they serve to further support the continuation of memantine drug trials in humans. Further questions to be addressed regarding memantine are the stimulation of synapse formation and excitatory synapse as well as excitatory synaptic transmission.

### Memantine effects in patients with fragile X

An open-label memantine drug trial was conducted among six patients with FXS (Erickson et al., 2009). Memantine was administered once a day starting at 5 mg and increased every 2 weeks by an additional 5 mg until the presence of a clinical response or side effects appeared, 20 mg was the maximum dose. Routine check-ups, CGI severity tests, and baseline and post-treatment Aberrant Behavior Checklists (ABC) were used to analyze memantine's effects on patients. The average for all six participants across the five categories of the ABC test, irritability, social withdrawal, stereotypy, hyperactivity, and inappropriate speech decreased from baseline scores to post-trial scores (Erickson et al., 2009).

Close examination of the patient specific data demonstrates benefits of memantine use in four out of the six patients. In patients 1 and 2, memantine significantly reduced irritability but in patients 5 and 6 irritability increased (Erickson et al., 2009). Patients 1, 2, 4 and 6 remained on other medications during this study, which may have affected memantine's effectiveness. Clinical data in psychiatric non-Alzheimer disease conditions often fail to support memantine as a monotherapy, this may be why patient five results included increased irritability (Sani et al., 2012) Memantine showed potential as a treatment for FXS however, the patient pool was too small therefore there is not enough evidence to know which future patients would respond best to this treatment.

## RETT syndrome and memantine

RTT was first described in 1954 by Dr. Andreas Rett. It was not until 1999 that a team at Baylor found *MECP2* on the X chromosome, when mutated, to cause RTT (Zoghbi et al., 1999). The X-linked gene *MECP2* encodes for methyl-CpG-binding protein 2 (MeCP2), which is expressed throughout the body (Weng et al., 2011). More and more studies are describing that MeCP2 regulates neuronal plasticity and that synaptopathy is a major component of the Rett phenotype (Weng et al., 2011).

The use of memantine in RTT deserves a thorough examination, due in part to findings that have shown that glutamate levels tend to be increased in RTT (Pan et al., 1999). Since above normal levels of glutamate can become toxic to the form and function of neurons, reducing its downstream effects could be promising therapeutically. Studies using male mice where the expression of *Mecp2* was prevented, demonstrated that *in vitro* application of memantine restored short-term plasticity as assayed by recovery of post-tetanic potentiation and paired-pulse facilitation in the hippocampus, the brain region critical for short-term plasticity (Weng et al., 2011). Further advancement of this drug or similar NMDA receptor antagonists in other preclinical trials, more specifically in behavioral and motor assays, would be of great interest to propel this drug class. Disease related long term potentiation (LTP) deficits may be due to increased tonic basal activation of post-synaptic NMDA receptors and are amenable to reversal by the weak NMDA receptor-blocking drug, memantine (Weng et al., 2011). Hippocampal slice cultures from wild-type mice and *Mecp2*-stop mice were incubated in a memantine solution. The wild-type slice cultures were not affected by memantine but the *Mecp2*-stop slice cultures were able to rescue LTP and post-tetanic potentiation. Memantine was capable of partially reversing the synaptic deficits caused by the loss of MeCP2. This data is supported by previous research which demonstrated LTP saturation effects have been overcome by memantine (Frankiewicz and Parsons, 1999).

Memantine has the potential to significantly impact future RTT treatments. While studies have not investigated memantine's abilities to reverse some of the symptoms of RTT or to delay disease progression, their findings in memantine's ability to partially restore plasticity thus far are significant.

## Conclusion

The non-competitive NMDA receptor antagonist, memantine has the potential to restore synaptic plasticity and aid in the treatment of diseases affecting learning and memory. Although memantine improves conditions in diseases it should be emphasized that memantine only improves upon symptoms thus far and that it does not presently reverse or cure any diseases. Until the cures for DS, RTT, and FXS are found, memantine research can lead to a better understanding of these diseases, their pharmacological interactions with other drugs, and aid in treating the symptoms that affect both learning and memory.

### Conflict of interest statement

The authors declare that the research was conducted in the absence of any commercial or financial relationships that could be construed as a potential conflict of interest.
